# Coronavirus disease 2019 (COVID-19) in Italy: features on chest computed tomography using a structured report system

**DOI:** 10.1038/s41598-020-73788-5

**Published:** 2020-10-14

**Authors:** Grassi Roberto, Fusco Roberta, Belfiore Maria Paola, Montanelli Alessandro, Patelli Gianluigi, Urraro Fabrizio, Petrillo Antonella, Granata Vincenza, Sacco Palmino, Mazzei Maria Antonietta, Feragalli Beatrice, Reginelli Alfonso, Cappabianca Salvatore

**Affiliations:** 1 Division of Radiodiagnostic, “Università Degli Studi Della Campania Luigi Vanvitelli”, Naples, Italy; 2 Radiology Division, “Istituto Nazionale Tumori IRCCS Fondazione Pascale – IRCCS di Napoli”, Naples, Italy; 3 Laboratory Medicine Unit, ASST Bergamo Est, Seriate, Italy; 4 Department of Radiology, ASST Bergamo Est, Seriate, Italy; 5 Department of Radiological Sciences, Diagnostic Imaging Unit, “Azienda Ospedaliera Universitaria Senese”, Siena, Italy; 6 Department of Medical, Oral and Biotechnological Sciences - Radiology Unit “G. D’Annunzio”, University of Chieti-Pescara, Chieti, Italy

**Keywords:** Diseases, Infectious diseases

## Abstract

To assess the use of a structured report in the Chest Computed Tomography (CT) reporting of patients with suspicious viral pneumonia by COVID-19 and the evaluation of the main CT patterns. This study included 134 patients (43 women and 91 men; 68.8 years of mean age, range 29–93 years) with suspicious COVID-19 viral infection evaluated by reverse transcription real-time fluorescence polymerase chain reaction (RT-PCR) test. All patients underwent CT examinations at the time of admission. CT images were reviewed by two radiologists who identified COVID-19 CT patterns using a structured reports. Temporal difference mean value between RT-PCRs and CT scan was 0.18 days ± 2.0 days. CT findings were positive for viral pneumonia in 94.0% patients while COVID-19 was diagnosed at RT-PCR in 77.6% patients. Time mean value to complete the structured report by radiologist was 8.5 min ± 2.4 min. The disease on chest CT predominantly affected multiple lobes and the main CT feature was ground glass opacity (GGO) with or without consolidation (96.8%). GGO was predominantly bilateral (89.3%), peripheral (80.3%), multifocal/patching (70.5%). Consolidation disease was predominantly bilateral (83.9%) with prevalent peripheral (87.1%) and segmental (47.3%) distribution. Additional CT signs were the crazy-paving pattern in 75.4% of patients, the septal thickening in 37.3% of patients, the air bronchogram sign in 39.7% and the “reversed halo” sign in 23.8%. Less frequent characteristics at CT regard discrete pulmonary nodules, increased trunk diameter of the pulmonary artery, pleural effusion and pericardium effusion (7.9%, 6.3%, 14.3% and 16.7%, respectively). Barotrauma sign was absent in all the patients. High percentage (54.8%) of the patients had mediastinal lymphadenopathy. Using a Chest CT structured report, with a standardized language, we identified that the cardinal hallmarks of COVID-19 infection were bilateral, peripheral and multifocal/patching GGO and bilateral consolidation with peripheral and segmental distribution.

## Introduction

In December 2019, health authorities in Wuhan, China, recognized a cluster of acute respiratory disease of unknown etiology; the infection produced by the virus was called coronavirus 2019 (COVID-19) and can be extent through human to human contact^1,2^.

As of 24 March 2020, over 417,000 cases of COVID-19 have been confirmed worldwide, having been diagnosed in 168 territories in several countries including Western and South-Eastern pacific regions, European regions, Eastern Mediterranean regions as well as many states in the America^3,4^. In Europe, Italy is the country most affected and with the highest number of deaths^4^.

The mean value of incubation period is estimated to be 5.2 days^5^. Evidence shows that virus transmission can occur during the incubation period in asymptomatic patients. In addition, high sputum viral loads were found in the recovery phase in patients with new pneumonia infected with COVID-19^6^.

The COVID-19 diagnosis is established using reverse transcription real-time fluorescence polymerase chain reaction (RT-PCR) test performed on the respiratory tract or blood specimens.

Recent results have revealed the efficiency of some imaging methods in the management of COVID-19 disease. The chest X-ray examination, although not offering highly specific findings, provided a first overview of the patients, especially in the emergency room, and can direct the differential diagnosis between COVID-19 infection and other pathologies involving pulmonary parenchyma. Bandirali et al.^7^ reported that 100 of 170 (59%) chest x-rays (mean patient age 57 ± 16 years) had abnormalities highly suspicious for COVID-19 pneumonia. Involvement was bilateral in all cases: in 54% of patients, the involvement was symmetrical.

Furthermore, chest X-ray at the patient's bed, in hospitalized patients and in intensive care, is a valid tool for the pneumonia evolutionary monitoring^8^. Chest radiography typically shows patchy or diffuse asymmetric airspace opacities, similar to other causes of coronavirus pneumonias. The chest ultrasound (POCUS—Point-Of-Care UltraSound), performed by the intensivists at the patient's bed, can also represent a monitoring tool to evaluate the effectiveness of the prono-supination maneuvers^9^. In this sense, the systematic application of POCUS can reduce the use of diagnostic imaging resources, including also personnel exposed to the danger of contagion and help integrate therapies especially in critically ill patients^10^. On the other hand, the ultrasound scan itself requires prolonged contact between the operator and the patient, and therefore a series of contraindications.

Computed tomography (CT) examination was used to evaluate the grade and the extension of the viral pneumonia by COVID-19^11–13^. Although CT exams are routinely used for monitoring lung involvement, and several publications attempted to show that CT could differentiate COVID-19 from other viral pneumonias, the field is highly debated and several radiological organizations not have the CT recommended as a routine screening tool in the COVID-19 pneumonia identification^14–18^.

However, the diagnosis of viral pneumonia based on chest CT may indicate isolation and plays an important role in the management of patients with suspected SARSCoV-2 infection^12,13^. Radiologists took their attention on the main CT findings: ground-glass opacity (GGO), consolidation, presence of nodules and lesion distribution (unilateral or bilateral involvement, single or multiple lobes, etc.)^19^. The presence of GGO with bilateral distribution with or without consolidation was reported as the main CT features in patients affected by COVID-19 infection^12–21^. However, with the increase of the cases and of the investigations, a multiplicity of interesting CT features were found including crazy paving pattern, reversed halo sign, etc.^22–25^.

However, the detailed CT findings of COVID-19 have been reported in only a small number of articles in the literature^12–14^ without a structured report system and a standardized language to describe the CT signs. A standardized COVID-19 reporting language could improve communication with referring providers and could have the potential to enhance efficiency and aid in management of patients during this pandemic^25^.

We analyzed the chest CT images performed at the time of admission of 134 patients with suspicious SARS-CoV-2 infection in order to evaluate the main CT features by COVID-19 using a structured report system.

## Materials and methods

### Patient characteristics

In relation to the ongoing epidemic emergency, the Institutional review board (IRB) of “Bergamo Est” approved the study and waived written informed consent for this retrospective study that evaluated de-identified data and involved no potential risk to patients. All methods were carried out in accordance with relevant guidelines and regulations. Our cohort was composed of 134 (43 women and 91 men; 68.8 years of mean age—range, 29–93 years) subjected to the nucleic acid amplification test of the respiratory tract or blood specimens using RT-PCR test for suspicious COVID-19, between February 23, 2020, and March 5, 2020. The virus investigation for etiological diagnosis were executed by the current gold standard test in the clinical laboratory of ASST Bergamo Est (Seriate, Italy).

Patient characteristics were reported in Table 1.

**Table 1:** Demographic characteristics and CT findings of 134 Patients with Suspicious COVID-19 viral pneumonia.

Age (year)	Positive for COVID-19		Negative for COVID-19		*p* value
Mean	69.3		61.6		0.11
Range	29–93		43–81		

	Tot	%	Tot	%	*p* value
**Sex, no. (%) of patients**					
Male	89	70.6	2	25.0	**0.02**
Female	37	29.4	6	75.0	
**GGOs—consolidation—nodules presence**					
Presence of GGOs with consolidation	89	70.6%	1	12.5%	**<** **0.01**
Presence of GGOs without consolidation	33	26.2%	1	12.5%	> 0.05
Presence of consolidation without GGOs	4	3.2%	0	0.0%	> 0.05
Absence of GGOs and consolidation	0	0.0%	6	75.0%	**≪** **0.001**
Nodules	10	7.9%	2	25.0%	> 0.05
Septal thickening	47	37.3%	1	12.5%	> 0.05
Air bronchogram sign	50	39.7%	0	0.0%	> 0.05
Crazy paving pattern	95	75.4%	0	0.0%	**≪** **0.001**
“Reversed halo” sign	30	23.8%	0	0.0%	> 0.05
Pleural effusion	18	14.3%	0	0.0%	> 0.05
Pericardium effusion	21	16.7%	1	12.5%	> 0.05
Mediastinal lymphadenopathy	69	54.8%	1	12.5%	> 0.05
Increased trunk diameter of the pulmonary artery (diameter > 29 mm)	8	6.3%	0	0.0%	> 0.05
Barotrauma sign	0	0.0%	0	0.0%	> 0.05

*p* value was evaluated for continuous variable by Mann Whitney test and by Chi square test with Yates correction for categorical ones. The *p* values reported in bold were considered significant.

### CT technique

CT scan was performed at the time of patient admission in hospital. Two CT scanners (CT 128 slice Ingenuity of Philips, Amsterdam—Netherlands and CT 128 slice Optima 660 of GE Healthcare, Chicago, Illinois, United States) were used for all chest CT examinations. Conventional CT was performed with the patient in the supine position during end-inspiration. Chest CT protocol parameters for both scanners were described in Table 2.

**Table 2:** Chest CT protocols.

Parameter	High resolution protocol with CT 128 slice PHILIPS INGENUITY	High resolution protocol with CT 128 slice GE OPTIMA 660
Slice thickness	1 mm	1.25 mm
Slice increment	1 mm	1.25 mm
Pitch	0.94	1.35
Rotation time	0.5 s	0.5 s
Field of view	411,0 mm	500 mm
Voltage	120 kV	120 kV
mAs modulation	100–200 mA	120–400 mA

Every chest CT examination was evaluated by two double blind radiologists; the radiologists had 10 and 7 years’ experience in interpreting chest CT.

### CT review

**Figure 1:**
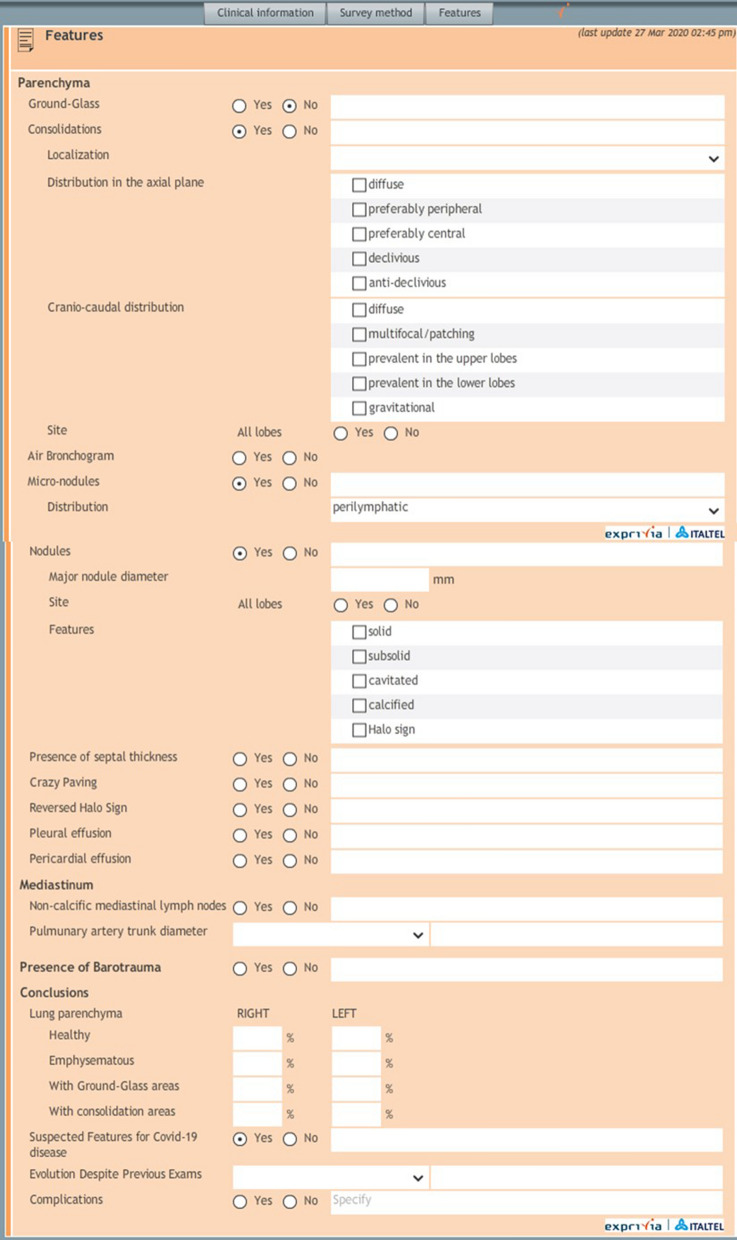
Structured report.

All chest CT examinations were reviewed using a structured report defined by Italian Society of Medical Radiology and Interventional Radiology (SIRM, Milan, Italy) in collaboration with the Exprivia Healtcare company (Bari, Italy) (Fig. 1).

The structured report includes, for the radiological signs section, a targeted, systematic and comprehensive description of all abnormalities and a description of the features that are relevant to the suspected pathology. Main CT features included in the report are the extension, distribution and localization of GGO and consolidations, air bronchogram sign, septal thickening, crazy paving pattern, “reversed halo” sign, nodules, pleural effusion, pericardium effusion, presence of mediastinal lymphadenopathy, diameter of the main pulmonary artery (more or less than 29 mm) and of the segmental arterial vessels, barotrauma sign. The radiologists, who were blinded to RT-PCR results, defined the presence of the above mentioned signs using the structured report. Rate of patients with positive results for COVID-19 pneumonia at CT scan was compared to the rate of patients with positive finding at RT-PCR.

We sought to identify the CT features of lesions more suggestive of COVID-19 based on the chest CT findings reported in the structured reports in order to define the cardinal hallmarks.

### Statistical analysis

Continuous data were expressed in terms of mean value and standard deviation and range. Categorical data are expressed as counts and percentages. Mann Whitney test was use to verify differences statistically significant between groups of continuous variables. Chi square test was used to assess statistically differences between percentage values among groups.

*p* value < 0.05 was considered significant for all tests.

All analyses were performed using Statistics Toolbox of Matlab R2007a (The Math-Works Inc., Natick, MA, United States).

## Results

### CT and RT-PCR performance results

Mean value of temporal difference between RT-PCRs execution and CT scan was 0.18 days ± 2.0 days. 120/134 (89.6%) cases subjected to the RT-PCR and CT scan in a temporal window of ± 2.0 days.

**Figure 2:**
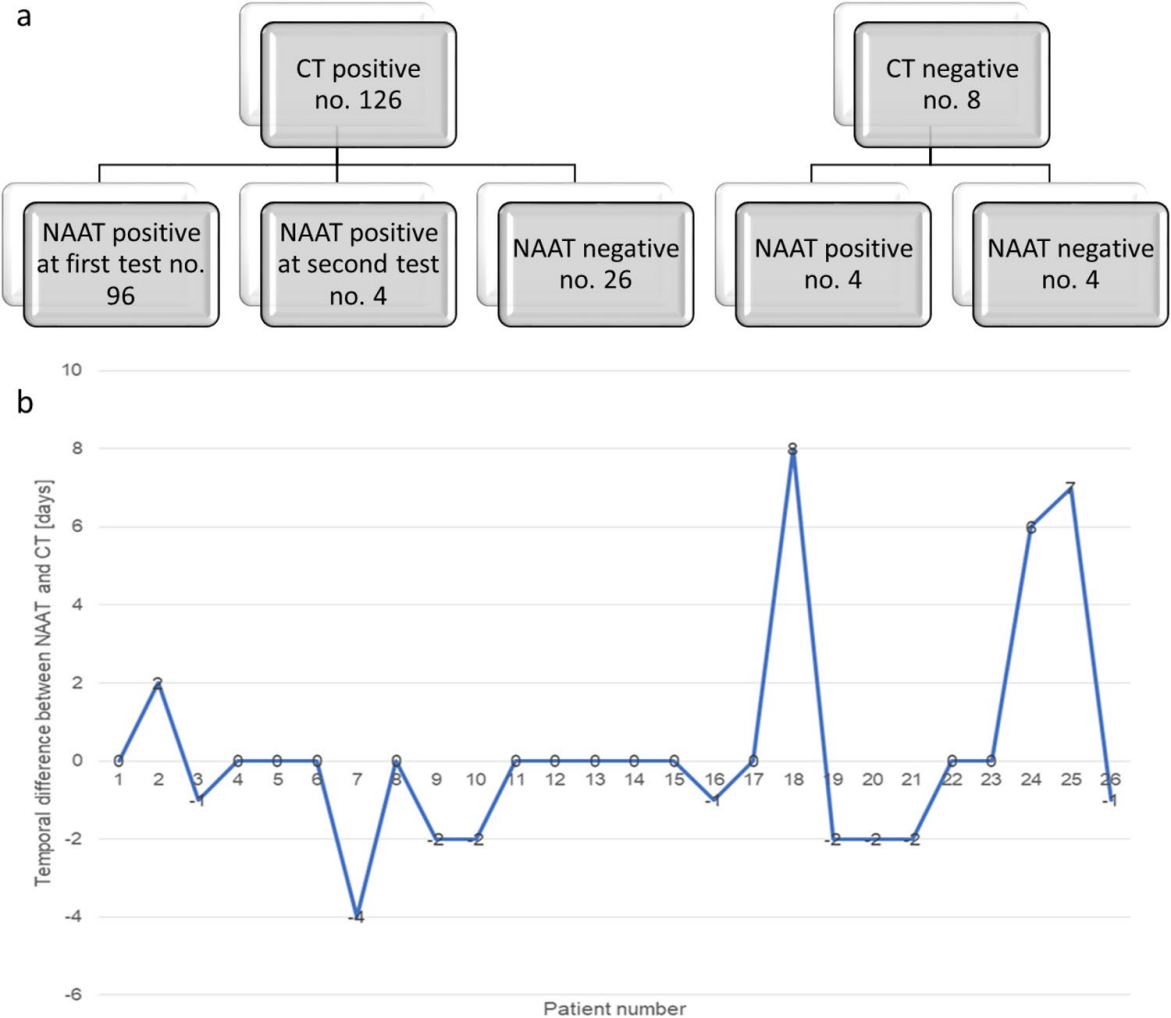
(**a**) Flowchart shows difference between positive results at RT-PCR test and positive findings at CT for COVID-19 viral pneumonia; (**b**) temporal difference between RT-PCR execution and CT scan for patients with positive diagnosis at CT scan and negative diagnosis at RT-PCR test.

CT findings were positive for viral pneumonia in 126 of 134 (94.0%) patients (Fig. 2a) while COVID-19 was diagnosed at RT-PCR in 104 of the 134 (77.6%) patients. The difference between two COVID-19 prevalence rate in this cohort was statistically significant with a *p* value < 0.01 at Chi square test.

In Fig. 2b, we highlighted the temporal difference in days between the positive CT diagnosis with negative RT-PCR results. Three cases had a temporal difference that ranges from 6 to 8 days that could explain the negative results at RT-PCR.

### More frequent CT features

Time mean value to complete the structured report by radiologist was 8.5 min ± 2.4 min.

**Figure 3:**
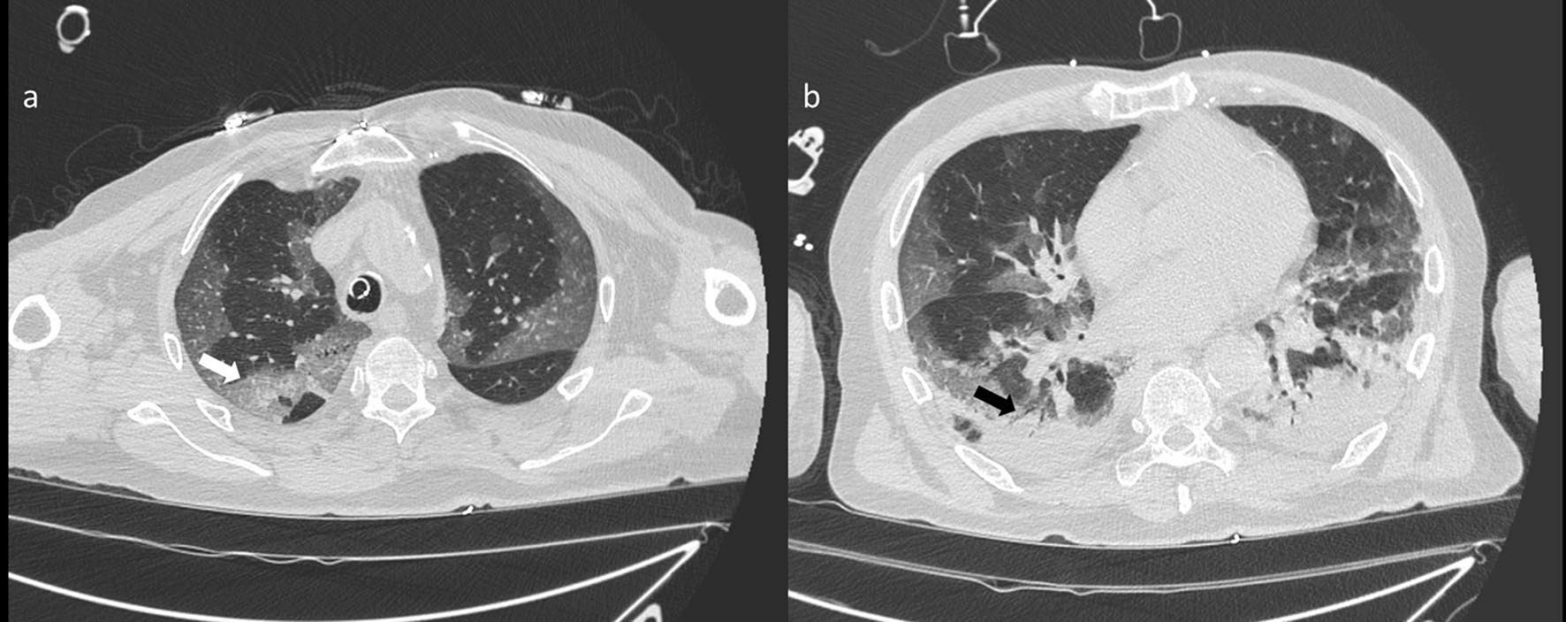
(**a**) CT scan shows bilateral areas of GGO involving upper lobes with prevalent peripheral distribution. In the right upper lobe there is a superimposed reticular pattern (white arrow). The patient is intubated as shown by the presence of the endotracheal tube; (**b**) CT scan shows areas of consolidation in peripheral subpleural region of the lower lobes with air bronchogram (black arrows).

GGO and consolidations were the two main signs of COVID-19 infection on CT images (Fig. 3). CT showed multiple irregular areas of GGOs or consolidation or both in 126 of the 134 (94.0%) patients. In the remaining eight (6.0%) patients, GGOs or consolidation were seen on 2 cases (Table 1). GGO is the cardinal hallmark in the patient with positive CT diagnosis for COVID-19 (Table 3): it was present in 122 on 126 cases (96.8%).

**Table 3:** GGO characteristics in patients with positive CT diagnosis for COVID-19 viral pneumonia.

GGO characteristics (no. 122 patients)	Tot	%
**Extension**		
Monolateral	13	10.7%
Bilateral	109	89.3%
**Distribution**		
Peripheral–central	76	62.3%
Diffuse	20	16.4%
Peripheral	17	13.9%
Diffuse–declivous	2	1.6%
Peripheral–declivous	3	2.5%
Peripheral–central–declivous	1	0.8%
Peripheral–central–declivous	1	0.8%
N/A	2	1.6%
**Localization**		
Multifocal/patching	81	66.4%
Diffuse	24	19.7%
Segmental	8	6.6%
Multifocal/patching–diffuse	3	2.5%
Segmental–multifocal/patching	2	1.6%
N/A	4	3.3%
**Site**		
Multiple lobes	112	91.8%
RLL	4	3.3%
LUL	2	1.6%
RUL	1	0.8%
LLL	1	0.8%
N/A	2	1.6%

Note. RUL = right upper lobe, RLL = right lower lobe, LLL = left lower lobe, LUL = left upper lobe, N/A = not available.

**Figure 4:**
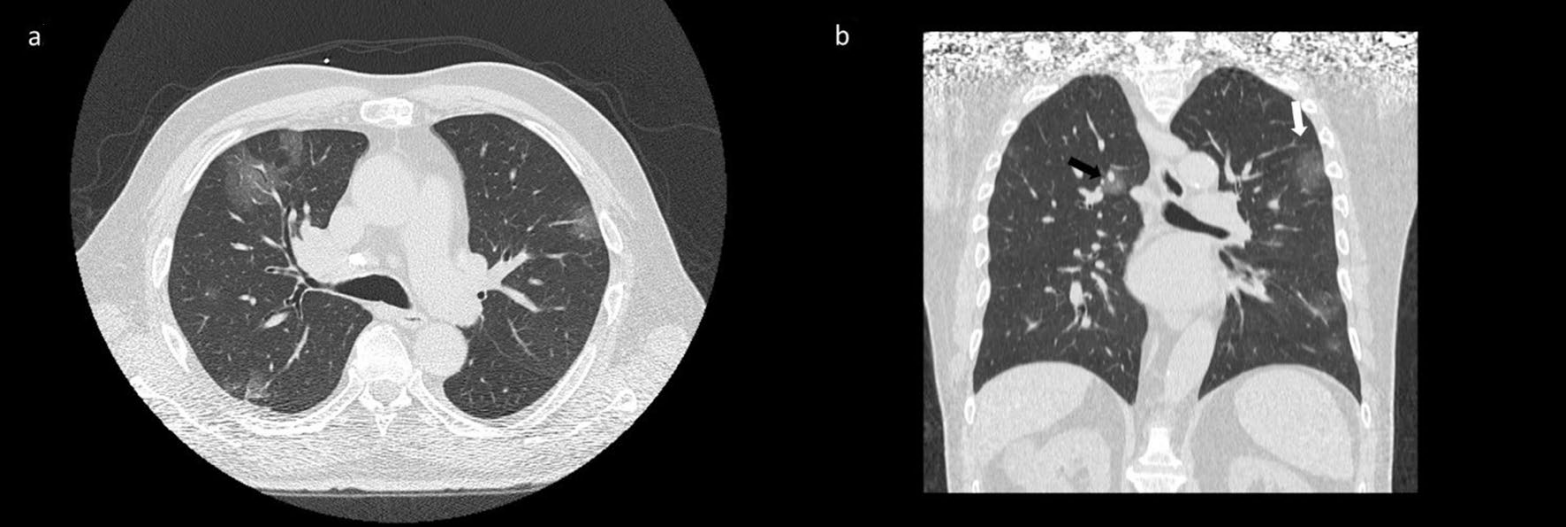
(**a**) Axial CT scan thought upper lobes shows bilateral areas of GGO with central (black arrow) and peripheral (white arrow) distribution as depicted in the CT coronal reformation (**b**).

**Figure 5:**
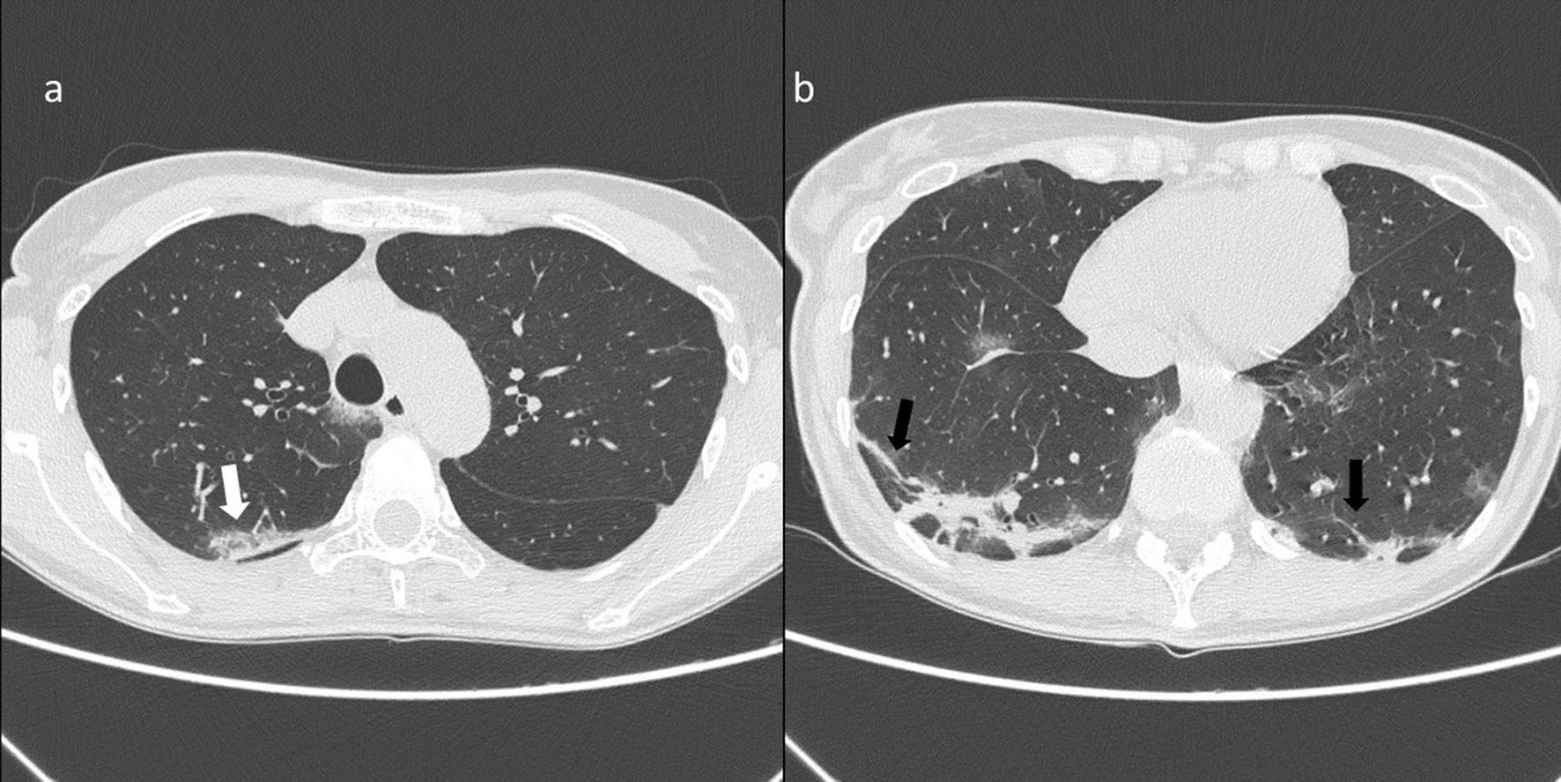
(**a**) CT scan shows focal consolidation in the subpleural area of the right upper lobe (white arrow); (**b**) CT scan shows bilateral areas of consolidation in the lower lobes with peripheral distribution and reticular pattern with the presence of fibrous stripes (black arrows).

The presence of GGOs was statistically significant respect to the group with negative findings at CT for COVID-19 with a significant *p* value (< 0.01) at Chi square test. GGOs were predominantly bilateral in 109/122 (89.3%) patients, peripheral in 98/122 (80.3%) patients, multifocal/patching in 86/122 (70.5%) patients (Fig. 4, Table 3). Consolidation disease was predominantly bilateral in 78/93 (83.9%) patients, peripheral in 81/93 (87.1%) patients, segmental in 44/93 (47.3%) patients (Fig. 5, Table 4).

**Table 4:** Consolidation characteristics in patients with positive CT diagnosis for COVID-19 viral pneumonia.

Consolidation characteristics (no. 93 patients)	Tot	%
**Extention**		
Monolateral	13	14.0%
Bilateral	78	83.9%
N/A	2	2.2%
**Distribution**		
Peripheral	36	38.7%
Peripheral–declivous	25	26.9%
Peripheral–central	19	20.4%
Diffuse	6	6.5%
Declivous	3	3.2%
Diffuse–declivous	2	2.1%
Peripheral–central–declivous	1	1.1%
N/A	1	1.1%
**Localization**		
Segmental	44	47.3%
Multifocal/patching	25	26.9%
Diffuse	17	18.3%
Multifocal/patching–diffuse	1	1.1%
N/A	6	6.5%
**Site**		
Multiple lobes	78	83.9%
RLL	6	6.5%
LUL	4	4.3%
RUL	4	4.3%
LLL	1	1.1%

*RUL* right upper lobe, *RLL* right lower lobe, *LLL* left lower lobe, *LUL* left upper lobe, *N/A* not available.

CT studies showed that disease predominantly affected multiple lobes (in case of GGOs presence in 112/122 patients (81.8%), in case of consolidation in 78/93 patients (83.9%) (Tables 3 and 4).

**Figure 6:**
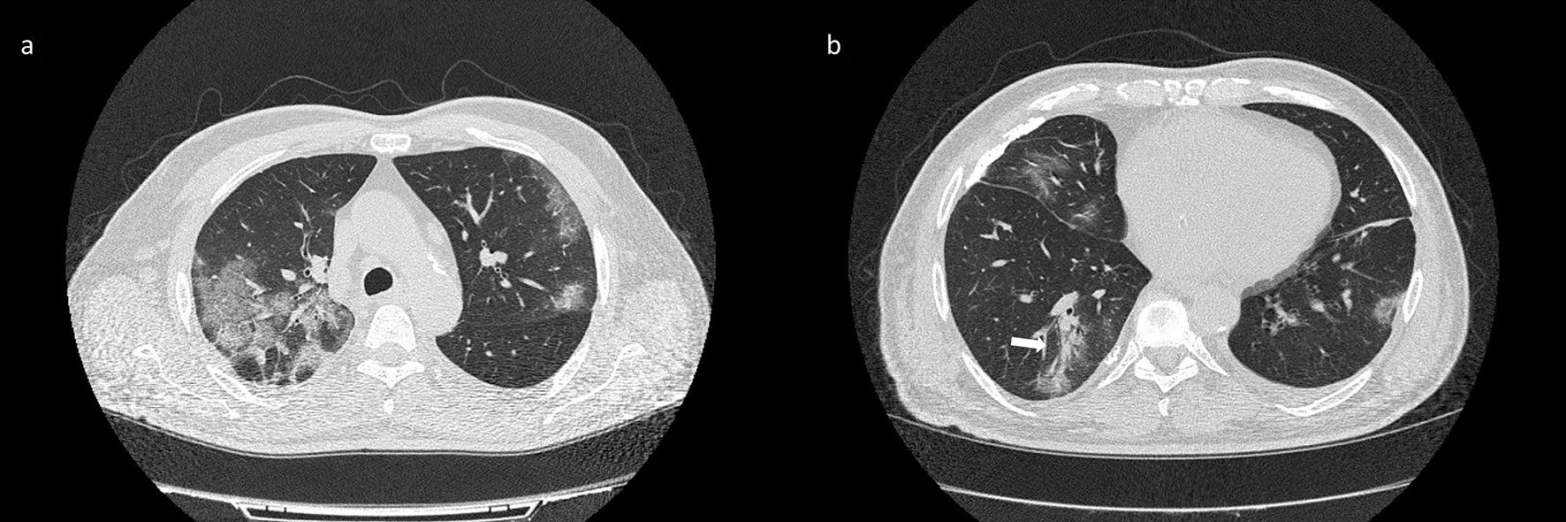
(**a**) CT scan through upper lobes shows reticular pattern superimposed on the background of GGO with patchy distribution identifying a crazy paving pattern; (**b**) lower CT scan shows multifocal GGOs and consolidation with air bronchogram in the right lower lobe (withe arrow).

**Figure 7:**
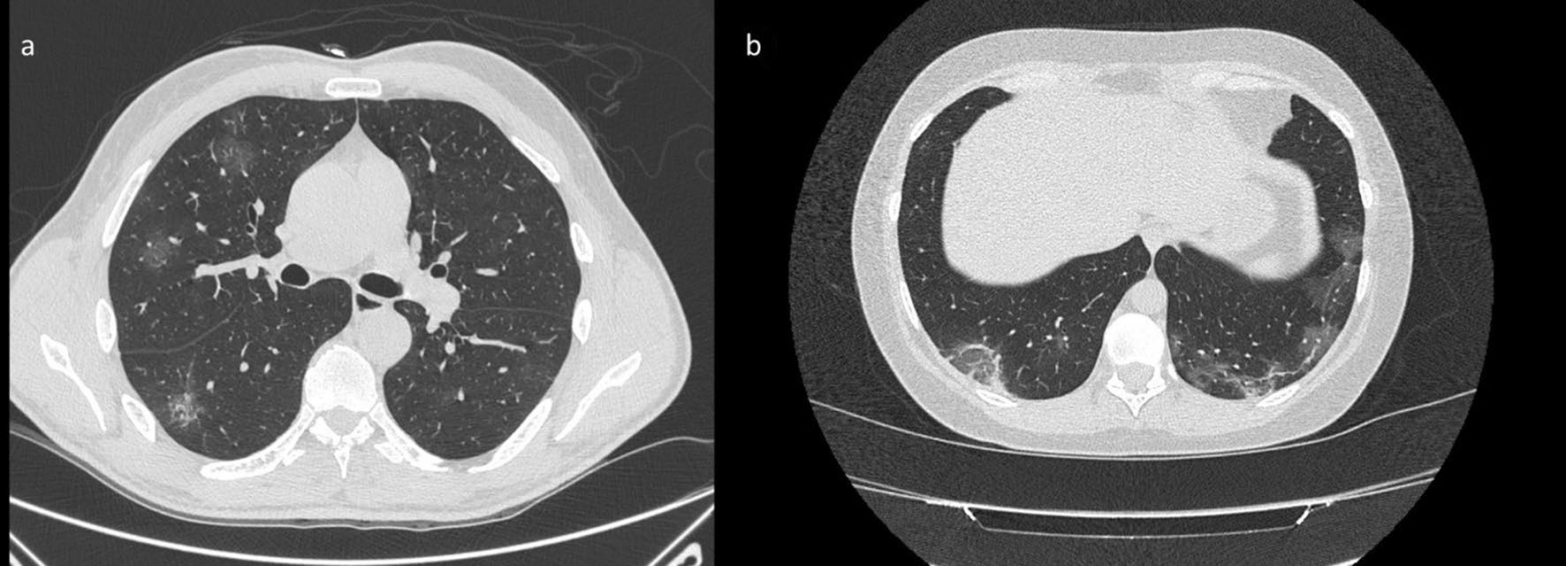
(**a**) CT scan shows multiple areas of GGO in the right upper and lower lobes with typical rounded morphology; (**b**) CT scan thought lower lobes shows bilateral involvement of posterior subpleural region of both lungs with areas of GGO and mild reticular pattern. Note the presence of “reversed halo sign” in the posterior segment of the right lower lobe.

We noted additional significant signs of COVID-19 lesions on CT images. CT showed a crazy-paving pattern (Fig. 6) in 95/126 (75.4%) patients with a significant *p* value (≪ 0.001) at Chi square test, the septal thickening in 47/126 (37.3%) patients, the air bronchogram sign (Fig. 6) in 50/126 (39.7%) patients, the “reversed halo” sign (Fig. 7) in 30/126 (23.8%) patients (Table 1).

Mediastinal non calcified lymphadenopathies with a short diameter equal or greater than 1 cm were found in 69/126 (54.8%) patients.

### Less frequent CT features

Pulmonary nodules were found in 10/126 (7.9%) patients (3 patients had nodules with a halo sign and 5 had solid or sub-solid nodules without a halo sign, Table 1); increased trunk diameter of the pulmonary artery in 8/126 (6.3%) patients, pleural effusion in 18/126 (14.3%) and pericardium effusion in 21/126 (16.7%) patients. Barotrauma signs were absent in all the patients.

## Discussion and conclusions

Routine screening CT for the identification of COVID-19 pneumonia is currently not recommended by most Radiologic Societies (Italian Society of Medical and Interventional Radiology, American College of Radiology^14^, Royal College of Radiologists^16^, Royal Australian and New Zealand College of Radiology reports^15^ and Canadian Association of Radiologists^17^ ) that declare that “CT never can be used in screening programs and that CT as any other diagnostic methods, cannot predict COVID-19 onset”.

Moreover, Zhiliang et al.^26^, reported that the 29.2% of patients those COVID-19 positive closed contacts never developed any symptoms or changes on chest CT. The other presented changes on CT, but only 21% developed symptoms during their hospital course and none of them developed severe disease. This suggests that a high percentage of COVID-19 carriers are asymptomatic. In 112 cases with confirmed COVID-19 diagnosis at RT-PCR, chest CT images of asymptomatic and symptomatic patients among the passengers and crew of the Diamond Princess cruise ship were analyzed. Of the asymptomatic cases, 54% showed CT signs of pneumonia while 80% of the symptoms had positive CT. Asymptomatic patients tended to show patterns with predominant appearance of the GGO while symptomatic patients tended to have lung thickenings more frequently^27^.

Several publications have described CT imaging features in patients affected by COVID-19, the evolution of these features over time, and the radiologists performance to differentiate COVID-19 from other viral infections^12,20,22,28^. These studies have shown that COVID-19 often occurs on CT images with peripheral GGO and nodular or mass-like GGO with a distribution bilateral and multilobar^29^. Guan et al.^30^ showed that the most common chest CT patterns were GGO (56.4%) and bilateral patchy shadowing (51.8%).

However, additional imaging findings have been reported including linear, curvilinear or perilobular opacities, consolidation and diffuse GGO, which can mimic various pathological processes such as other infections, inhalational exposures, and drug toxicities^31,32^. Moreover, Valente et al.^33^ evidenced the importance to report also the incidental findings, not linked to the pneumonia, including peripheral pulmonary artery aneurysms or incidental pulmonary nodules.

The first report of patients with COVID-19 described bilateral lung involvement on initial chest CT in 40 of 41 patients, with a consolidative pattern seen in patients in the Intensive Care Unit (ICU) and a predominantly GGO pattern in patients who were not in the ICU^12^. An investigation in 21 individuals with confirmed COVID-19 described abnormal findings in 86% of chest CT, with bilateral lung involvement in 88.9%^15^. Multifocal GGO and consolidations were reported in 57% and 29%, respectively, with a peripheral lung tendency^28^.

Some studies reported that chest CT findings could precede positivity on RT-PCR test. In the present study, CT findings resulted positive for viral pneumonia in 126 of 134 (94.0%) patients while COVID-19 was diagnosed at RT-PCR in 104 of them (77.6%); the difference between two COVID-19 prevalence rate in this cohort was statistically significant. However, the 22 patients with positive findings at the CT and negative RT-PCR test could have a pneumonia different by COVID-19 (influenza A and B or bacterial pneumonia). The findings visible on CT cannot allow for a safe differentiation of COVID-19 pneumonia from other forms of pneumonia. Moreover, the findings visible at CT could be due, in some patients, at a different timing among the two examinations. These results were according to literature reporting that RT-PCR sensitivity ranges from 42 to 71%^34,35^, and that an initially negative RT-PCR may take up to 4 days to convert in a patient with positive COVID-19 diagnosis^34^. The reported sensitivities and specificities of CT for COVID-19 vary widely (60 to 98% and 25 to 53%, respectively)^34–38^, probably related to the studies retrospective nature, including lack of strict diagnostic imaging criteria and procedural to confirm the infection. The CT positive and negative predictive value (PPV and NPV) for COVID-19 diagnosis are estimated at 92% and 42%, respectively, in a population with high pretest disease probability^35^. Ai et al.^38^ reported that the sensitivity of chest CT in suggesting COVID-19 was 97% based on positive RT-PCR results. In patients with negative RT-PCR results, 75% (308/413) had positive chest CT findings; the specificity, PPV, NPV and accuracy reported were 25%, 65%, 83% and 68% respectively. The relatively low NPV reported in these studies^36,37^ suggests that CT may not be an adequate COVID-19 screening test in earlier stages of the disease and the relatively low PPV suggests that CT may not be a valid decision making. Furthermore, the safe use of CT to study COVID-19 patients is logistically demanding and can overwhelm the available resources. Even with proper cleaning protocols, healthcare professionals and CT scanners could become infection vectors for other vulnerable patients requiring imaging.

The goal of structured reporting in the setting of COVID-19 pneumonia is to provide a standardized language in the description of the CT findings decreasing reporting variability allowing the immediacy of the report, reducing waiting times, facilitating the result understanding by other specialists, reducing the uncertainty in reporting findings potentially attributable to this infection, thereby allowing better integration into clinical decision making. While we do not currently recommend the use of CT screening for COVID-19 pneumonia, we suggest using a standardized language when specifically asked to address whether or not findings of COVID-19 pneumonia may be present on CT images and propose language that could be placed in the radiologist report.

The use of structured report allowed to identify the main CT features in this cohort of 134 patients subjected to CT scan for COVID-19 suspicion at time of the admission. Our results, according to the recent literature, showed that the disease predominantly affects multiple lobes without any lobar prevalence. Multifocal areas of GGO, with or without consolidations (96.8%), were the main CT features in patients with COVID-19 infection. GGOs were predominantly bilateral (89.3%) with peripheral (80.3%) and patchy (70.5%) distribution. Consolidation disease was predominantly bilateral (83.9%) with peripheral (87.1%) and segmental (47.3%) distribution. Moreover, we noted additional significant CT signs of COVID-19 infection such as crazy-paving pattern, seen in 75.4% of patients; septal thickening seen in 37.3% of patients, air bronchogram sign in 39.7% of patients and “reversed halo” sign in 23.8% of cases. Discrete pulmonary nodules, increased trunk diameter of the pulmonary artery, pleural effusion can be found but in a low non-significant percentage of cases (7.9%, 6.3%, 14.3%, respectively). The pericardium effusion was reported in the 16.7% of patients; this could determine cardiac injury that is a common condition among patients hospitalized with COVID-19, associated with higher risk of in-hospital mortality, as reported by Shi et al.^39^. Barotrauma sign was absent in all the patients. In this cohort, differently from what is reported in the literature^20,24^, high percentage of suspicious patients for COVID-19 had mediastinal lymphadenopathy greater than 1 cm in short axis diameter (54.8%).

The main limitation of the present study is the nature retrospective and monocentric of the study conducted on a cohort of symptomatic hospitalized patients from an area of high epidemiological risk and with a high pre-test probability of COVID-19 infection.

In conclusion, the use of a structured report could support the management^40^ of interstitial pneumonia from COVID-19 identifying the cardinal hallmarks of COVID-19 infection on CT imaging represented by bilateral, multifocal GGOs with peripheral and patchy distribution and bilateral consolidations with prevalent peripheral and segmental distribution. Other CT findings such as “crazy-paving” pattern, septal thickening, air bronchogram and “reversed halo” sign must be listed.

## Key results

1. GGO and consolidations were the two main signs of COVID-19 lesions on CT images.
2. GGOs were predominantly bilateral with a peripheral and multifocal/patching distribution.
3. Consolidation disease was predominantly bilateral, peripheral and segmental.

## Required summary statement

The use of a structured report could support the management of interstitial pneumonia from COVID-19 identifying the cardinal hallmarks of COVID-19 infection on CT imaging.
