# Author Correction: Coronavirus disease 2019 (COVID-19) in Italy: features on chest computed tomography using a structured report system

**DOI:** 10.1038/s41598-021-81863-8

**Published:** 2021-02-15

**Authors:** Roberto Grassi, Roberta Fusco, Maria Paola Belfiore, Alessandro Montanelli, Gianluigi Patelli, Fabrizio Urraro, Antonella Petrillo, Vincenza Granata, Palmino Sacco, Maria Antonietta Mazzei, Beatrice Feragalli, Alfonso Reginelli, Salvatore Cappabianca

**Affiliations:** 1 Division of Radiodiagnostic, “Università Degli Studi Della Campania Luigi Vanvitelli”, Naples, Italy; 2 Radiology Division, “Istituto Nazionale Tumori IRCCS Fondazione Pascale – IRCCS di Napoli”, Naples, Italy; 3 Laboratory Medicine Unit, ASST Bergamo Est, Seriate, Italy; 4 Department of Radiology, ASST Bergamo Est, Seriate, Italy; 5 Department of Radiological Sciences, Diagnostic Imaging Unit, “Azienda Ospedaliera Universitaria Senese”, Siena, Italy; 6 Department of Medical, Oral and Biotechnological Sciences ‑ Radiology Unit “G. D’Annunzio”, University of Chieti-Pescara, Chieti, Italy

Correction to: *Scientific Reports* https://doi.org/10.1038/s41598-020-73788-5, published online 14 October 2020

The original version of this Article contained errors in the spelling of the authors Roberto Grassi, Roberta Fusco, Maria Paola Belfiore, Alessandro Montanelli, Gianluigi Patelli, Fabrizio Urraro, Antonella Petrillo, Vincenza Granata, Palmino Sacco, Maria Antonietta Mazzei, Beatrice Feragalli, Alfonso Reginelli & Salvatore Cappabianca which were incorrectly given as Grassi Roberto, Fusco Roberta, Belfiore Maria Paola, Montanelli Alessandro, Patelli Gianluigi, Urraro Fabrizio, Petrillo Antonella, Granata Vincenza, Sacco Palmino, Mazzei Maria Antonietta, Feragalli Beatrice, Reginelli Alfonso & Cappabianca Salvatore.

These errors have now been corrected in the PDF and HTML versions of the Article.

